# Assessment of Overall Survival in Glioma Patients as Predicted by Metabolomic Criteria

**DOI:** 10.3389/fonc.2019.00328

**Published:** 2019-05-10

**Authors:** María L. Gandía-González, Sebastián Cerdán, Laura Barrios, Pilar López-Larrubia, Pablo G. Feijoó, Alexis Palpan Jr., José M. Roda, Juan Solivera

**Affiliations:** ^1^Department of Neurosurgery, Hospital Universitario La Paz, Madrid, Spain; ^2^Institute of Biomedical Research “Alberto Sols” CSIC/UAM, Madrid, Spain; ^3^Department of Statistics CSIC, Madrid, Spain; ^4^Department of Neurosurgery, University Hospital Reina Sofía, Córdoba, Spain

**Keywords:** classification decision tree, glioma, metabolomic profile, high resolution proton magnetic resonance spectroscopy, overall survival

## Abstract

**Objective:** We assess the efficacy of the metabolomic profile from glioma biopsies in providing estimates of postsurgical Overall Survival in glioma patients.

**Methods:** Tumor biopsies from 46 patients bearing gliomas, obtained neurosurgically in the period 1992–1998, were analyzed by high resolution ^1^H magnetic resonance spectroscopy (HR- ^1^H MRS), following retrospectively individual postsurgical Overall Survival up to 720 weeks.

**Results:** The Overall Survival profile could be resolved in three groups; Short (shorter than 52 weeks, *n* = 19), Intermediate (between 53 and 364 weeks, *n* = 19) or Long (longer than 365 weeks, *n* = 8), respectively. Classical histopathological analysis assigned WHO grades II–IV to every biopsy but notably, some patients with low grade glioma depicted unexpectedly Short Overall Survival, while some patients with high grade glioma, presented unpredictably Long Overall Survival. To explore the reasons underlying these different responses, we analyzed HR-^1^H MRS spectra from acid extracts of the same biopsies, to characterize the metabolite patterns associated to OS predictions. Poor prognosis was found in biopsies with higher contents of alanine, acetate, glutamate, total choline, phosphorylcholine, and glycine, while more favorable prognosis was achieved in biopsies with larger contents of total creatine, glycerol-phosphorylcholine, and myo-inositol. We then implemented a multivariate analysis to identify hierarchically the influence of metabolomic biomarkers on OS predictions, using a Classification Regression Tree (CRT) approach. The CRT based in metabolomic biomarkers grew up to three branches and split into eight nodes, predicting correctly the outcome of 94.7% of the patients in the Short Overall Survival group, 78.9% of the patients in the Intermediate Overall Survival group, and 75% of the patients in the Long Overall Survival group, respectively.

**Conclusion:** Present results indicate that metabolic profiling by HR-^1^H MRS improves the Overall Survival predictions derived exclusively from classical histopathological gradings, thus favoring more precise therapeutic decisions.

## Introduction

Gliomas are the most frequent primary brain tumors, currently managed through surgical resection, radiotherapy, and chemotherapy (1) approaches, but leading inevitably to large disability and mortality outcomes. The selection of the recommended therapeutic intervention in each case relies in estimates overall survival (OS) based commonly in histopathological and genetic criteria. However, current assessments of OS entail considerable uncertainties, limiting concomitantly more precise, effective, and personalized therapies. On these grounds, exploring additional criteria to improve OS predictions acquires vital relevance to improve treatment outcomes in glioma patients.

Histopathological and immunohistochemical criteria have classically provided the basis for the initial WHO classification of gliomas in grades I-IV (2, 3) determining, in general terms, the OS estimate and the recommended therapeutic intervention. More recently, the 2016 WHO classification of central nervous system tumors added an important collection of molecular signatures, restructuring the original histopathological classification of gliomas to include subgroups with specific genetic profiles (4). Although these refinements considerably improved the precision in the treatment prescribed, as well as our knowledge of glioma physiopathology and classification, the limited reproducibility of histopathological evaluations lead, not unfrequently, to imprecise histopathological classification and unreliable OS predictions at the individual level (5–7).

Magnetic Resonance Imaging approaches have been currently used to assess OS of gliomas. Briefly, radiomic parameters including surface area (8), shape features (9), tumor, and necrosis volumes, necrosis-tumor ratio (10) have been used to evaluate OS. However, these studies became many times limited to short OS periods as they evaluated only glioblastoma multiforme cases.

The metabolomic profiles of gliomas are able to provide an additional source of information to improve OS predictions, evaluating the down-stream metabolic alterations of aberrant cellularity and gene expression (11). Magnetic Resonance Spectroscopy (MRS) has been shown to be well-endowed to provide the metabolic profile of gliomas both *in vivo* and *in vitro* (12, 13). Briefly, *in vivo* ^1^H MRS revealed non-invasively, important hallmarks of cancer, including alterations in pH homeostasis (14), energy related (15), and phospholipid metabolites (16, 17), an ensemble of valuable metabolic fingerprints to classify high grade (HGG) or low grade gliomas (LGG) (16, 18). Moreover, the metabolic profiles determined by ^1^H MRS *in vivo* reached considerable clinical prognostic relevance (19–22). Alternatively, complementary *in vitro* HR-^1^H MRS approaches have proved to be able to resolve a larger number of metabolites than *in vivo* ^1^H MRS, thus increasing the size of the metabolome investigated, the number of potential alterations detected, and their influence on the tumoral phenotype, at the expense of the more invasive *in vitro* methodology (23, 24). However, the predictive role of the metabolomic profiles obtained by HR-^1^H MRS in providing OS estimates, received considerably less attention.

On these grounds, we aimed here to provide a pilot study evaluating OS estimates derived from metabolomic biomarkers as detected by HR-^1^H MRS, using a retrospective database of human glioma biopsies.

## Materials and Methods

### Glioma Patients and Tumor Biopsies

**Figure 1:**
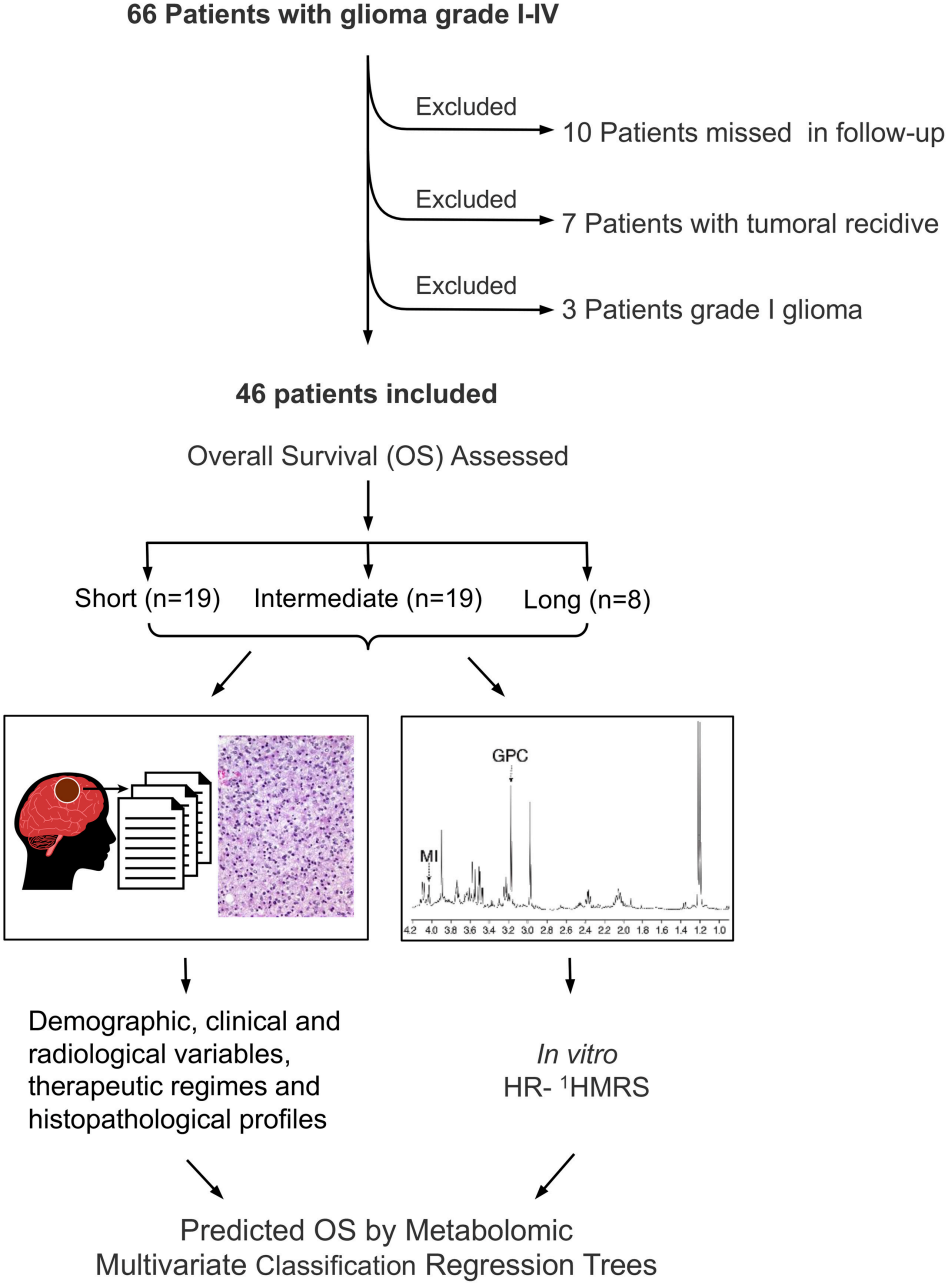
Overview of glioma patient selection and implemented approach.

This study was approved by the Ethics Committee of Clinical Research from the University Hospital La Paz (http://www.madrid.org/cs/Satellite?language=es&pagename=HospitalLaPaz/Page/HPAZ_home) and carried out following their recommendations. All subjects gave written informed consent in accordance with the Declaration of Helsinki. We retrospectively reviewed the database of the Neurosurgery Department of the University Hospital La Paz, selecting 66 consecutive patients with glioma (grades I-IV) who underwent neurosurgery during the period 1992–1998 (Figure 1). Briefly, solid parts of glioma tumors were extracted from the brain without the use of bipolar coagulation and divided into two adjacent and similar portions, one of them used for HR-^1^H MRS analysis and the other for histopathological diagnosis, following available WHO criteria (25). Tumor characteristics were evaluated by two independent radiologists and classified according to size, localization, and eloquence (26). Patients lost in follow up (*n* = 10) and those undergoing surgery for recurrence (*n* = 7) were excluded from further analysis. Grade I gliomas (*n* = 3) were also excluded because of their well-known physiopathological differences with the other glioma grades (27, 28). We then recovered the individual demographic, clinical, histopathological and *in vitro* spectroscopic ^1^H NMR features and gathered the OS information on postsurgical outcomes of these patients, including relevant clinical symptoms, adjuvant therapies, and OS time. No missing data for the variables of interest were found in these patients.

### Histopathological Criteria

Histopathological grading of the biopsies was provided by the Anatomopathology Department of the Hospital, following standard WHO criteria (25), and archived until used in this study.

### HR-^1^H MRS

The ^1^H MRS biopsy was immediately frozen in liquid nitrogen (−169°C) in the operating room of the Hospital and stored at −82°C until transferred in a liquid nitrogen container to the Institute of Biomedical Research CSIC/UAM for further processing and NMR analysis. Briefly, biopsies were reduced to powder in a previously chilled (methanol/dry ice) mortar, extracted with 6% perchloric acid, neutralized with KOD, lyophilized and resuspended in D_2_O (99.9% D) for ^1^H MRS analysis (12). HR-^1^H MRS spectra of biopsy extracts were acquired at 8.4 Tesla (360.13 MHz, pH 7.2, 22°C) in a Bruker AM-360 spectrometer equipped with a commercial ^1^H selective probe using 5-mm tubes and 0.5 ml of tissue extract. Acquisition conditions were: 90° pulses, 16.9 s total cycle time and 16,384 data points acquired in the time domain during of 1.901 s. The intensity of the residual water resonance was further reduced using a 5 s presaturating pulse centered on the water frequency. Prior to Fourier transformation, the free induction decays were zero-filled up to 128 K and multiplied by an exponential function resulting in 0.5 Hz artificial line broadening in the transformed spectrum. Further spectral processing, including phase and baseline corrections were performed by the same operator. Chemical shifts were referred to the methyl signal of TSP (2,2′-3,3′ tetradeutero trimethyl-sylil propionate sodium salt) at 0 ppm as an internal reference.

The following metabolites (resonances used in quantification, number of protons originating the resonance, multiplicity) could be consistently identified in the high resolution proton spectrum (29): Valine (1.09 ppm, 3H, d), Lactate (1.35 ppm, 3H, d), Alanine (1.45 ppm, 3H, d), Acetate (1.93 ppm, 3H, s), N-acetyl-aspartic acid (2.01 ppm, 3H, s), Gamma-amino butyric acid (2.31 ppm, 2H, t), Glutamate (2.43 ppm,2H, m), Glutamine (2.45 ppm, 2H, m), Aspartic acid (2.80 ppm, 2H, dd), Creatine (3.05 ppm, 3H, s) and Phosphocreatine (3.055 ppm, 3H, s), free Choline (3.20 ppm, 9H, s), Phosphorylcholine (3.22ppm, 9H, s), Glycerophophorylcholine (3.25 ppm, 9H, s), Taurine (3.45 ppm, 2H, t), Glycine (3.55 ppm, 2H, s), and Myo-inositol 4.07 ppm, 1H, dd). For every one of these metabolites, lorentzian curves were fitted to the most conveniently resolved proton resonances, and the resulting integral divided by the total number of protons of the corresponding metabolite (6). These values were further standardized by the sum of all the measured metabolites in the HR-^1^H NMR spectra and expressed as a molar percentage (18, 30). Assignments were performed with the aid of chemical shift values reported in the literature (29, 31) and confirmed when necessary by the addition of authentic standards.

### Statistical Methods

Statistical analyses were performed using the IBM SPSS Statistics 24 package as implemented on an Intel-PC platform, operating under Windows 10 environment. Univariate statistical approaches provided means and standard errors for the molar fractions of every metabolite. To investigate statistical dependences between clinical features and groups of OS, we used asymptotic chi-square with Monte Carlo exact probability tests, and to test the differences of means within each metabolic variable through the OS groups, we used the ANOVA test and Student *t*-tests. Finally, to explore the hierarchical contribution of individual HR-^1^H MRS biomarkers to the three groups of OS, we implemented a multivariate Classification Regression Tree (CRT) (32), classifying automatically the database using hierarchical nodes and branches, selecting step-wise the optimal discriminant biomarker for each split from the collection of available HR-^1^H MRS variables. The dependent variable was OS, using Chi-squared Automatic Interaction Detection (CHAID) as a growing method to provide automatically the optimal splits in every branch. Finally, we used these results to generate a Classification-Confusion Matrix (CCM), summarizing the correct and incorrect classifications provided by the metabolomics CRT, yielding the global percentage of correct classifications. Statistical significance in the ANOVA and multivariate analysis was defined as *p* = 0.05, considering confidence intervals higher than 95%.

## Results

### Glioma Database

**Figure 2:**
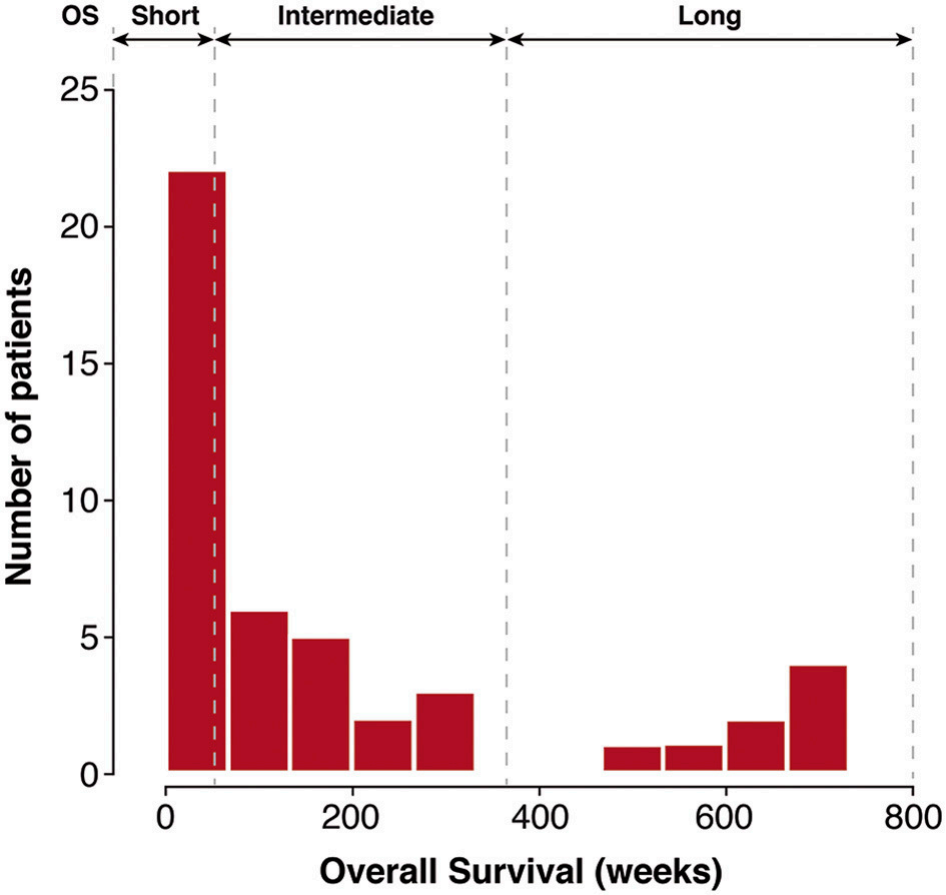
Histogram of OS in glioma patients after surgical resection at the Neurosurgery Department, University Hospital La Paz, in the period 1995–1998. Patient groups depicting Short-OS (1–52 w), Intermediate-OS (53–364 w) and Long-OS (>364 w) are separated by the dotted lines.

We investigated 46 patients (23 males, 23 females) with a median age of 49 years, presenting the following glioma grade distribution; Grade II (11 cases), Grade III (16 cases), and Grade IV (19 cases). The database of glioma patients (Figure 2) showed two clearly separated groups by OS, either depicting a Long Overall Survival (Long-OS) that survive more than 364 weeks (w), or less. The latter group, including patients with a wide range of survival (1–364 w), was further divided in two groups using the median of survival as a cut-point, resulting in Short Overall Survival (Short-OS) patients (1–52 w), or Intermediate Overall Survival (Intermediate-OS) patients (53–364 w), respectively.

Demographic, clinical, and radiological variables, therapeutic regimes and histopathological profiles of these groups are summarized in Table 1. A Chi-square Monte Carlo test was performed to compare the differences in independent clinical variables among the three survival categories. Radiotherapy, histopathologic grade, age, and localization showed statistically significant influence on OS.

**Table 1:** Clinical features and overall survival of glioma patients.

**Feature**		**Overall survival**			**Total patients *n* (% column)**	**Chi-square (montecarlo sig.)**
		**Short *n* (% row)**	**Intermediate *n* (% row)**	**Long *n* (% row)**		
Age	<25 y	0 (0.0%)	5 (62.5%)	3(37.5%)	8 (17.4%)	0.01
	25–54 y	5 (31.3%)	7 (43.8%)	4(25%)	16 (34.8%)	
	>54 y	14 (63.6%)	7 (31.8%)	1 (4.5%)	22 (47.8%)	
Sex	Female	8 (34.8%)	11(47.8%)	4 (17.4%)	23 (50%)	0.74
	Men	11(47.8%)	8 (34.8%)	4 (17.4%)	23 (50%)	
Comorbility^a^	Yes	10 (34.5%)	13 (44.8%)	6 (20.7%)	29 (63%)	0.56
	No	9 (52.9%)	6 (35.3%)	2 (11.8%)	17 (37%)	
Localization^b^	A	2 (15.4%)	7 (53.8%)	4 (30.8%)	13 (30.2%)	0.04
	B	5 (41.7%)	6 (50%)	1 (8.3%)	12 (27.9%)	
	C	12 (66.7%)	3 (16.7%)	3 (16.7%)	18 (41.9%)	
Tumor volume^b^	Small	13 (39.4%)	15 (45.5%)	5 (15.2%)	33 (73.3%)	0.49
	Big	6 (50%)	3 (25%)	3 (25%)	12 (26.7%)	
Eloquency^b^	Yes	16 (53.3%)	10 (33.3%)	4 (13.3%)	30 (69.8%)	0.19
	No	3 (23.1%)	6 (46.2%)	4(30.8 %)	13 (30.2%)	
Resection	Partial/Complete	10 (52.6%)	5 (26.3%)	4 (21.1%)	19 (41.3%)	0.27
	Biopsy	9 (33.3%)	14 (51.9%)	4 (14.8%)	27 (58.7%)	
Radiotherapy	Yes	19 (54.3%)	12 (34.3%)	4 (11.4%)	35 (76.1%)	0.003
	No	0 (0%)	7 (63.6%)	4 (36.4%)	11 (23.9%)	
Chemotherapy	Yes	0 (0%)	1 (33.3%)	2 (66.7%)	3 (6.5%)	0.07
	No	19(44.2%)	18 (41.9%)	6 (14%)	43 (93.5%)	
Histopathology grade^c^	II	0 (0%)	6 (54.5%)	5 (45.5%)	11 (23.9%)	<0.001
	III	3 (18.8%)	10 (62.5%)	3 (18.8%)	16 (34.8%)	
	IV	16(84.2%)	3 (15.8%)	0 (0%)	19 (41.3%)	

a *Arterial hypertension, diabetes mellitus and/or pulmonary, renal, cardiac, oncologic or any severe disease*,

b *Classification of tumors according to (26)*.

c *Histopathologic grade according to (3)*.

### HR-^1^H MRS

**Figure 3:**
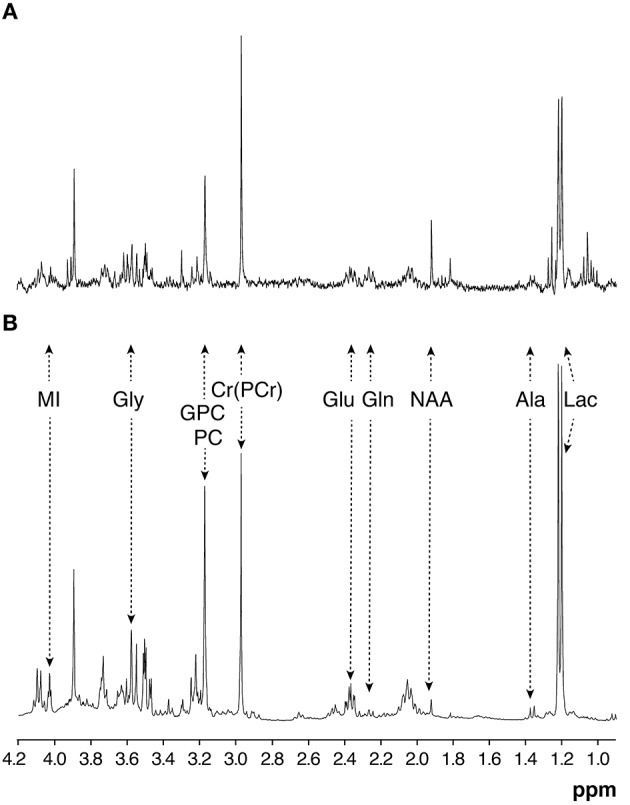
Representative HR-^1^H MRS (360.13 MHz, 22°C, pH 7.2) from extracts of two glioma grade II biopsies with very different OS outcomes. **(A)** 149 w. **(B)** 600 w. MI, myo-inositol; Gly, glycine; GPC, glycerolphosphorylcholine; PC, phosphorylcholine; Cr, creatine; PCr, phosphocreatine; Glu, glutamate; Gln, glutamie; NAA, N-acetyl-aspartic acid; Ala, alanine; Lac, lactate.

An illustrative example of underdetermined histopathological OS prediction is provided in Figure 3, showing representative HR-^1^H MRS spectra from extracts of glioma biopsies obtained from two young male patients, assigned the same histopathological Grade II, but resulting in very different OS. Despite both patients underwent complete surgical resection without adjuvant radio- or chemotherapy, the patient represented in Figure 3A, survived <3 years (149 w), while the patient represented by Figure 3B survived more than 11 years (605 w).

Interestingly, HR-^1^H MRS spectra of these biopsies disclosed remarkable differences, particularly the relative increases in myo-inositol (MI) and glycerol-phosphorylcholine (GPC) in Figure 3B. These findings suggested that HR-^1^H MRS analysis of biopsy extracts could contribute additional OS criteria to those normally obtained from general histopathological classification, thus prompting further HR-^1^H MRS analyses of the database.

### Univariate Statistics

**Figure 4:**
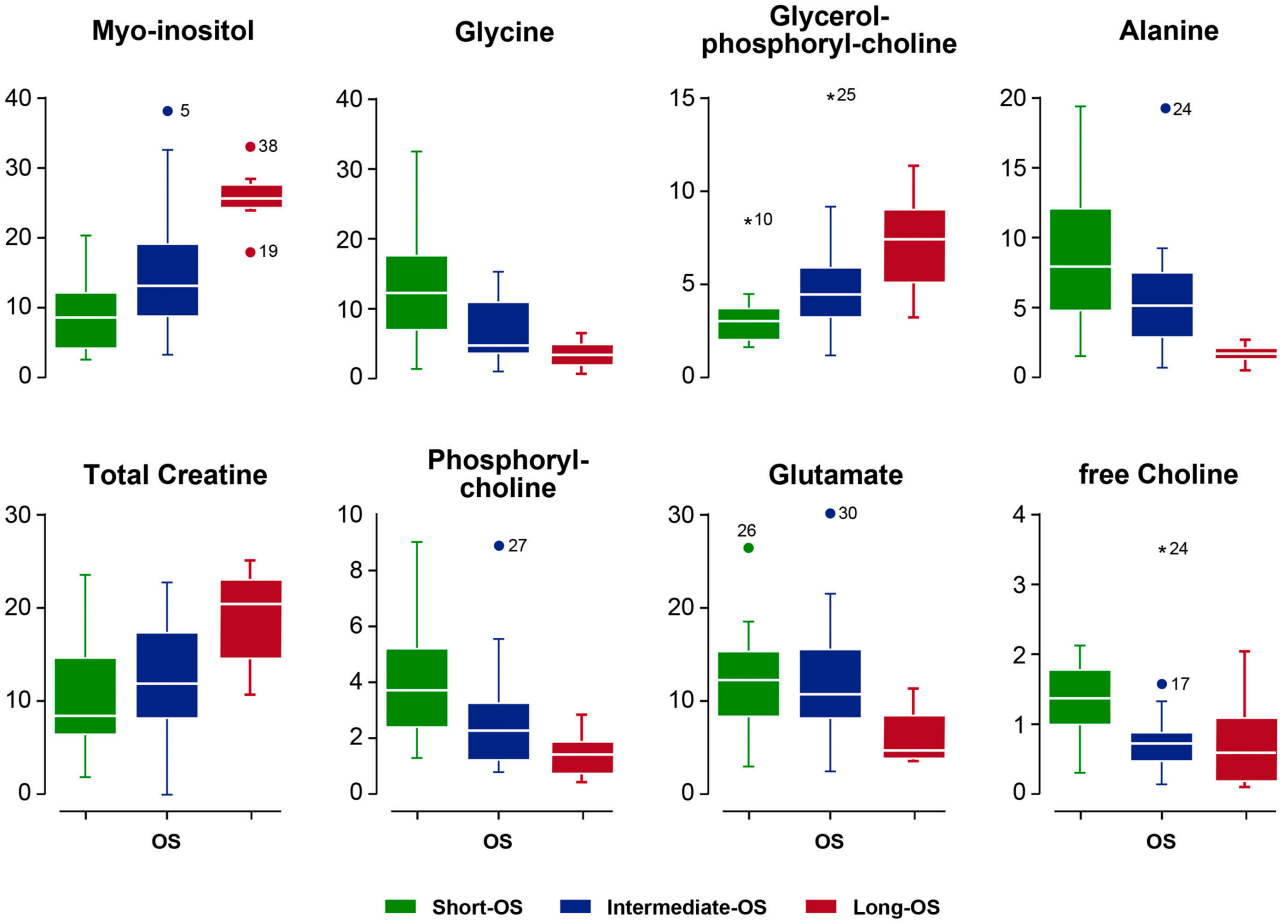
Box-plots of metabolite molar ratios in extracts from glioma biopsies with Short-OS (green), Intermediate-OS (blue) and Long-OS (red). In each box, the central mark (white line) indicates the median, and the bottom and top edges refer to the 25-th and 75-th percentiles, respectively. The upper and lower limits of the box extend to the most extreme data points not considered outliers.

OS was well-reflected in the metabolic profiles obtained, with evident relationships between OS and specific metabolite changes (Figure 4). Table 2 provides a more detailed analysis of the relationship between OS and specific metabolite molar fractions, highlighting the discriminant power in OS of each metabolite (*F*-value) and its statistical significance (*p*-value) as derived from ANOVA tests.

**Table 2:** General linear model analysis of OS in glioma patients as revealed by the metabolic profile determined by HR-^1^H MRS.

**Metabolite**	**Short (*n* = 19)**	**Intermediate (*n* = 19)**	**Long (*n* = 8)**	**Total**	***F*^b^**	***p***
**OVERALL SURVIVAL^a^**						
**MI**	**9.03 ± 1.26**	**14.75 ± 2.14**	**25.72 ± 1.51**	**14.30 ± 1.36**	**15.10**	**<0.001**
**Ala**	**8.47 ± 1.12**	**5.67 ± 0.95**	**1.67 ± 0.24**	**6.13 ± 0.70**	**13.65**	**<0.001**
**GPC**	**3.17 ± 0.34**	**5.17 ± 0.72**	**7.18 ± 0.97**	**4.70 ± 0.42**	**9.44**	**<0.001**
**Gly**	**13.38 ± 1.89**	**6.95 ± 1.03**	**3.61 ± 0.68**	**9.03 ± 1.05**	**9.37**	**<0.001**
**PC**	**4.17 ± 0.48**	**2.84 ± 0.48**	**1.43 ± 0.28**	**3.14 ± 0.32**	**8.36**	**0.001**
**tCr**	**13.36 ± 1.10**	**15.03 ± 1.19**	**20.84 ± 1.63**	**15.35 ± 0.81**	**6.53**	**0.003**
**Ac**	**4.49 ± 0.97**	**3.09 ± 0.81**	**0.94 ± 0.17**	**3.30 ± 0.55**	**5.72**	**0.006**
**fCho**	**1.33 ± 0.13**	**0.86 ± 0.17**	**0.73 ± 0.24**	**1.03 ± 0.10**	**4.65**	**0.01**
**Glu**	**12.20 ± 1.27**	**12.09 ± 1.56**	**6.04 ± 1.16**	**11.08 ± 0.91**	**3.62**	**0.03**
Gln	15.47 ± 1.19	18.76 ± 1.38	18.51 ± 1.30	17.36 ± 0.81	2.02	0.14
Val	2.34 ± 0.47	1.60± 0.43	1.45 ± 0.23	1.88 ± 0.27	0.896	0.42
Asp	0.77 ± 0.14	0.70 ± 0.14	0.50 ± 0.12	0.69 ± 0.09	0.584	0.56
NAA	3.35 ± 0.94	4.57 ± 0.95	4.55 ± 0.94	4.06 ± 0.57	0.544	0.58
GABA	1.25 ± 0.31	0.92 ± 0.15	1.17 ± 0.33	1.10 ± 0.15	0.483	0.62
Tau	5.69 ± 0.79	6.17 ± 0.71	4.95 ± 0.63	5.76 ± 0.45	0.416	0.66
tCho	8.67 ± 0.57	8.88 ± 0.76	9.33 ± 1.02	8.87 ± 0.42	0.145	0.86

a *Results are given as mean ± standard error of mean*.

b *A Box Cox transform was performed on the data before running ANOVA analysis. Ac, Acetate; Ala, Alanine; Asp, aspartic acid; fCho, free choline; GABA, gamma-aminobutyric acid; Gln, glutamine; Glu, glutamate; Gly, glycine; GPC, glycerophosphocholine; MI, myo-inositol; NAA, N-acetyl-aspartic acid; PC, phosphorylcholine; Tau, taurine; tCr, total creatine; Val, valine. Bold characters indicate p < 0.05*.

Briefly, OS increased with increasing levels of MI, GPC, and total Creatine (tCr), and decreased with increasing levels of alanine (Ala), free choline (fCho), glutamate (Glu), phosphorylcholine (PC), and glycine (Gly). The remaining metabolites detected by HR-^1^H NMR, including acetate (Ac), glutamine (Gln), valine (Val), aspartate (Asp), N-acetyl-aspartic acid (NAA), Gamma-amino butyric (GABA), taurine (Tau), and total choline (tCho) were not found to influence significantly OS. The most powerful discriminators of OS were (Metabolite/ F value/ *p*-value) in decreasing order; MI/15.1/0.000, Ala/13.6/0.000, GPC/9.5/0.001, Gly/9.4/0.000, tCr/6.5/0.003, Ac/5.7/0.006, fCho/4.6/0.015, Glu/3.6/0.035.

We also investigated the relationship between histopathological grade and the molar fractions of metabolites detectable in extracts glioma biopsies using ANOVA tests (Table 3). Notably, the priority of metabolites providing optimal glioma grade discrimination, was different from the one yielding optimal OS discriminative power (Table 2). The following molar ratios of metabolites were found to provide optimal discriminant power between histopathological grades (metabolite/*F*-value/*p*-value); MI/21.7/0.000, PC/11.7/0.000, GPC/9.2/0.000, Ala/8.8/0.001, Gly/6.5/0.003, tCr/6.3/0.004, Glu 5.9/0.005 and Gln/3.5/0.040.

**Table 3:** General linear model analysis of the HR- ^1^H NMR metabolic profiles associated to different glioma histopathological grades.

	**IV**	**III**	**II**	**Total**	**F**	**p**
	***n* = 11**	***n* = 16**	***n* = 19**			
**HISTOPATHOLOGICAL GRADE^a^**						
**MI**	**8.41 ± 1.09^b^**	**13.94 ± 1.95**	**24.99 ± 2.3**	**14.3 ± 1.36**	**21.71^c^**	**<0.000**
**PC**	**4.36 ± 0.56**	**2.90 ± 0.36**	**1.40 ± 0.25**	**3.14 ± 0.32**	**11.74**	**<0.000**
**GPC**	**3.01 ± 0.19**	**5.32 ± 0.84**	**6.70 ± 0.87**	**4.70 ± 0.42**	**9.16**	**<0.000**
**Ala**	**8.36 ± 1.15**	**5.77 ± 1.09**	**2.82 ± 0.69**	**6.13 ± 0.7**	**8.85**	**0.001**
**Gly**	**12.74 ± 1.9**	**7.98 ± 1.42**	**4.14 ± 0.53**	**9.03 ± 1.05**	**6.54**	**0.003**
**tCr**	**13.54 ± 1.17**	**14.35 ± 1.31**	**19.94 ± 1.28**	**15.35 ± 0.81**	**6.30**	**0.004**
**Glu**	**13.85 ± 1.55**	**10.89 ± 1.32**	**6.59 ± 0.95**	**11.08 ± 0.91**	**5.90**	**0.005**
**Succ**	**1.36 ± 0.49**	**1.11 ± 0.2**	**0.58 ± 0.08**	**1.09 ± 0.22**	**3.99**	**0.03**
**Gln**	**14.95 ± 1.14**	**19.05 ± 1.31**	**19.05 ± 1.67**	**17.36 ± 0.81**	**3.47**	**0.04**
fCho	1.23 ± 0.13	1.05 ± 0.21	0.67 ± 0.16	1.03 ± 0.1	3.10	0.05
Val	2.32 ± 0.48	1.97 ± 0.49	0.98 ± 0.11	1.88 ± 0.27	2.51	0.09
Asp	0.88 ± 0.16	0.63 ± 0.12	0.47 ± 0.12	0.69 ± 0.09	1.93	0.16
Ac	3.75 ± 0.96	3.68 ± 0.98	1.95 ± 0.67	3.30 ± 0.55	1.49	0.24
Tau	6.09 ± 0.85	6.05 ± 0.74	4.77 ± 0.46	5.76 ± 0.45	0.42	0.66
tCho	8.60 ± 0.57	9.27 ± 0.86	8.76 ± 0.83	8.87 ± 0.42	0.24	0.79
GABA	1.03 ± 0.19	1.25 ± 0.36	1.02 ± 0.23	1.10 ± 0.15	0.23	0.80
NAA	4.14 ± 1.17	4.06 ± 0.86	3.94 ± 0.49	4.06 ± 0.57	0.01	0.99

a According to Louis et al. (3)

b *Results are given as mean ± standard error of mean*.

c *A Box Cox transform was performed on the data before running ANOVA analysis. Bold characters indicate p < 0.05*.

Together, these results show that relevant metabolites contribute with different strengths either to histopathological grading or to OS predictions.

### Classification Regression Trees (CRT)

**Figure 5:**
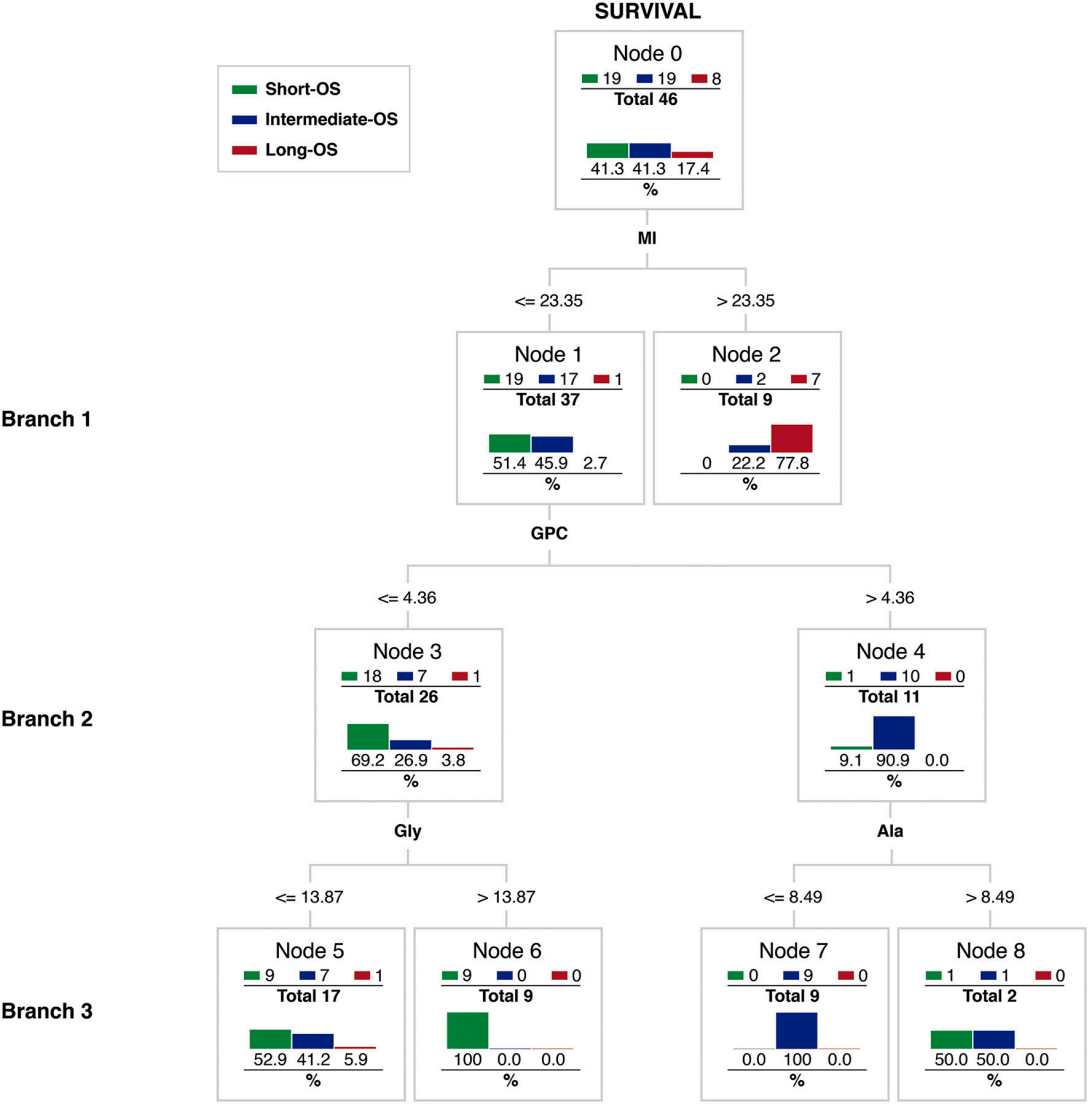
Classification Regression Tree (CRT) of glioma OS based in metabolomic biomarkers detectable by HR-^1^H MRS.

To investigate the hierarchical contribution of these metabolites to the OS observed, we implemented a multivariate CRT (Figure 5) (32). Starting with the complete patient database (Node 0), high MI levels (Branch 1) provided the most powerful biomarker to predict Long-OS survival within the three OS groups. MI levels ≤ 23.35 were found in 37 biopsies (Node 1). Of these, only one patient survived more than 7 years (Long-OS), while the rest of the patients depicted either Intermediate- (17 patients) or Short-OS (19 patients). In contrast, MI levels >23.35 were detected in nine biopsies (Node 2), of which seven (77.8%) depicted Long-OS, two showed Intermediate-OS, and none had a Short-OS, suggesting that high MI levels dismiss a Short-OS prediction.

Patients with MI levels ≤ 23.35 could be further split in two groups using GPC (Brach 2). Sixty-nine percent of the patients with GPC levels ≤ 4.36 (Node 3) were classified as Short-OS, while 91% of the patients with GPC > 4.36 (Node 4) were classified as Intermediate-OS. GPC provided thus a convenient biomarker to distinguish between Short- and Intermediate-OS estimates.

The metabolomic CRT grew beyond Branch 2, improving the classification using either Gly or Ala splits (Branch 3). All patients of Node 3 with Gly levels higher 13.87 (Node 6), depicted Short-OS, suggesting that high Gly levels are predictive of a negative outcome. Patients from Node 4, could be further stratified by their Ala levels. Those having Ala levels lower than 8.48 (Node 7) indicated Intermediate-OS (100%). Summarizing, the metabolomic CRT indicated dominant roles of MI, GPC, Ala, and Gly OS prediction of glioma patients.

Finally, we compared the number of correct predictions derived from the metabolomic CRT with those observed clinically, in the Classification-Confusion Matrix of Table 4. Out of 19 patients in the Short-OS group, the metabolomic approach classified correctly 18 patients. In the Intermediate-OS group, the metabolomic approach classified correctly 15 of 19 patients. Finally, out of the 8 patients identified with Long-OS, the metabolomic approach correctly classified 6 patients.

**Table 4:** Classification confusion matrix of correct/incorrect classifications of overall survival in patients bearing gliomas using metabolomic criteria.

**Observed overall survival (*n* = 46)**	**Predicted overall survival**			**Percent correct classifications**
	**Short**	**Intermediate**	**Long**	
Short (*n* = 19)	**18**^a^	1	0	**94.7**
Intermediate (*n* = 19)	4	**15**	0	**78.9**
Long (*n* = 8)	1	1	**6**	**75.0**
All patients (*n* = 46)				**84.7**

a *Numbers in bold indicate number and percentages of correct classifications. Growing Methods: CRT, dependent variable OS*.

In summary, the metabolomic classification reached defined OS predictions in all three groups, separating well the longer OS groups (Intermediate-OS and Long-OS). This entails considerable relevance, since the prediction of Long-OS in glioma patients remains currently a vital challenge for neurosurgeons, with important implications in the definition of the recommended therapeutic strategy.

## Discussion

### Previous OS Studies

The present study complements and extends earlier OS predictors based on the WHO classification (2–4) or *in vivo* MRI/MRS studies (19–21, 33–40), contributing a novel array of metabolomic biomarkers organized hierarchically by a multivariate CRT. Earlier studies implementing *in vivo* MRS/MRI approaches investigated mainly glioblastoma patients (Table 5), associating Short-OS to increases in PC (4) and tCho/NAA (20, 36–40, 42), and longer OS to higher contents in GPC (35). Additionally, Cho/Cr ratio has been proposed as a biomarker of cellular proliferation and prognosis (22). More recently, a correlation between 2-hydroxiglutarate and the IDH1 mutation (43), suggested that *in vivo* detection of 2-hydroxiglutarate could become a useful prognostic biomarker. However, routinely and regular detection *in vivo* of 2-hydroxyglutarate still remains an important technological challenge in most imaging centers, limiting wider applications. In summary, the present study contributes a larger cohort of patients examined by routinely available *in vitro* MRS, followed during a longer period of time, including also a collection of both Low Grade Gliomas (LGG) and High Grade Gliomas (HGG). Notably, some of the metabolomic biomarkers found valuable here in OS prediction, like MI or GPC (Table 2), are difficult to resolve, or not even detectable *in vivo*. Consequently, our results suggest that postsurgical HR-^1^H NMR analysis of extracted tumor biopsies may provide a useful complement to available *in vivo* MRS explorations when addressing OS predictions.

**Table 5:** Overview of literature correlating OS and MRS biomarkers.

**Authors & year**	**Patients**	**Glioma grade**	**Follow-up period^a^, ^b^**
Li et al. (39)	72	HGG^c^	17.2 m^a^
Reijneveld et al. (40)	14	LGG^d^	30 m (9–40)^b^
Hattingen et al. (41)	45	LGG	37 m (52.1–260.5)
Chang et al. (38)	143	LGG & HGG	n.s.
Yamasaki et al. (33)		HGG	26.1 m (6.5–83.8)
Steffen-Smith et al. (37)	39	HGG	7.1 m (1.6–61.6)
Quon et al. (36)	26	HGG	22.9 m (5–37)
Hattingen et al. (35)	32	HGG (recidives)	8.1 m
Tolia et al. (21)	12	HGG	n.s.
Steidl et al. (34)	37	HGG (recidives)	n.s.
Roldán et al. (20)	28	HGG	3–98 m
Present serie	46	LGG & HGG	14.9 m (0.24–170.4)

a *Median of the duration of the study in months*,

b *Parenthesis includes the range of the study in months (m)*,

c *HGG: High grade glioma*,

d *LGG: Low grade glioma, n.s.: not specified*.

### Metabolomic CRT

We implemented a CRT methodology to find, hierarchically, the best classification of OS estimates, using metabolomic criteria. These results complement the earlier decision tree of Li et al. (39), who considered age, MRI features (T_2_, T_1_, Volume Contrast Enhancement) and *in vivo* spectroscopic biomarkers (Lip+Lac, Cho/Cr, and Cr/NAA), as the main determinants predicting glioblastoma OS.

Our metabolomic CRT grew up to three branches. In the first branch, MI became the most robust biomarker of survival, with larger MI contents revealing longer survivals. Increased MI levels have been reported in inflammatory diseases as Alzheimer (41), renal failure (44), diabetes mellitus (44), and traumatic brain injury (45, 46), suggesting a universal role of this osmolite in pathophysiolological volume regulation. However, MI levels were reported previously to increase (47–49) or decrease (44) with increasing glioma grade *in vivo*. Present results reveal that higher MI levels are associated to longer OS, and lower tumoral grades (Table 3). Since MI occurs primarily in normal astrocytes (44), we hypothesize that the MI resonances detected in the tumor biopsies reveal the healthy astrocyte content within the tumor mass. Larger relative MI contents reveal indirectly relatively larger normal astrocyte populations and smaller tumoral cell burdens, supporting consequently longer OS (41, 50, 51).

The second hierarchical branch classifying the lower MI content group, is GPC, with higher GPC levels predicting longer OS. We, and others, have previously reported that relatively higher contributions of GPC and PC are associated to low and high grade gliomas, respectively (16, 52). Interestingly, GPC and PC levels are thought to reflect the balance between phospholipid degradation and phospholipid synthesis, respectively, with increased GPC levels revealing relatively a negative balance between synthesis and degradation, lower tumoral proliferation, and more prolonged survivals.

Gly and Ala provided the third branch of OS discrimination for patient groups with low or high levels of GPC, respectively. High Gly and Ala levels revealed poor prognosis, associated to shorter OS. Indeed, Ala and Gly levels previously reported as hypoxia and redox stress biomarkers (53), revealing tumoral progression to hypoxia, redox stress, and fatal energy failure. They can now be associated to shorter OS predictions.

### Limitations

The time span of biopsy collection in this study preceded some of the advances in the characterization of glioma genetics and their influence in malignancy and OS. This circumstance, and the long survival period investigated, precluded the use of genetic biomarkers validated later in the coverage of the present retrospective study. Thus, the correlations between metabolomic and genomic biomarkers of OS in glioma deserve further investigation. Finally, the number of patients involved in the present pilot study is admittedly small but sufficiently robust to support the use of metabolomic biomarkers detected by *in vitro* HR-^1^HMRS in OS predictions of postsurgical survival from glioma patients. A multicenter study to extend the number of patients and hospitals involved, is currently being implemented.

## Conclusion

We used a multivariate CRT to assess postsurgical OS predictions based in the ^1^H HR-MRS analysis of the metabolomic profile from neurosurgical biopsies of glioma patients. Present results show that the metabolic profiles of glioma biopsies constitute accurate and independent biomarkers of OS in glioma patients.

## Data Availability

All datasets generated for this study are included in the manuscript.

## Ethics Statement

This study was carried out in accordance with the recommendations of the Ethics Committee of the Hospital La Paz with the approval number PI-2097 with written informed consent from all subjects. All subjects gave written informed consent in accordance with the Declaration of Helsinki.

## Author Contributions

MG-G collected and integrated retrospective patient data, investigated survival patterns, and wrote the first draft. SC analyzed HR-^1^H NMR spectra. LB provided the univariate and multivariate statistical analyses. PL-L acquired HR-^1^H NMR spectra. PF and AP validated demographic, radiological, and histopathological assessments. JR performed many of the neurosurgical procedures providing integrated clinical information, and JS conceived the study and wrote the final draft with all authors commenting.

### Conflict of Interest Statement

The authors declare that the research was conducted in the absence of any commercial or financial relationships that could be construed as a potential conflict of interest.

## Abbreviations

Ac: Acetate
Ala: Alanine
Asp: aspartic acid
Cr: creatine
CCM: classification confusion matrix
CRT: Classification Regression Tree
d: doublet multiplicity
dd: doublet of doublets multiplicity
fCho: free choline
GABA: gamma-aminobutyric acid
GLM: General Lineal Model
Gln: glutamine
Glu: glutamate
Gly: glycine
GPC: glycerophosphocholine
GroPEtn: glycerophosphoethanolamine
HGG, high grade glioma ^1^H MRS: proton magnetic resonance spectroscopy
Lac: lactate
LGG: low grade glioma
Lip: lipid
m: multiplet
MRS: magnetic resonance spectroscopy
MI: myo-inositol
NAA: N-acetyl-aspartic acid
OS: overall survival
PC: phosphorylcholine
PCr: phosphocreatine
PDE: phosphodiester
PE: phosphoethanolamine
PtdCho: phosphatidylcholine
PtdEtn: phosphatidylethanolamine
PtdIns: phosphatidylinositol
s, singlet multiplicity SEM: Standard error of mean
t: triplet
Tau: taurine
tCho: total choline
tCr: total creatine
Val: valine
w: weeks.

## References

[1] Bush NA Chang SM Berger MS. Current and future strategies for treatment of glioma. Neurosurg Rev. (2017) 40:1–14. 10.1007/s10143-016-0709-8 27085859

[2] Biernat W. 2000 World Health Organization classification of tumors of the nervous system. Pol J Pathol. (2000) 51:107–114. 11247393

[3] Louis DN Ohgaki H Wiestler OD Cavenee WK Burger PC Jouvet A. The 2007 WHO classification of tumours of the central nervous system. Acta Neuropathol. (2007) 114:97–109. 10.1007/s00401-007-0243-4 17618441 PMC1929165

[4] Louis DN Perry A Reifenberger G von Deimling A Figarella-Branger D Cavenee WK. The 2016 World Health Organization classification of tumors of the central nervous system: a summary. Acta Neuropathol. (2016) 131:803–20. 10.1007/s00401-016-1545-1 27157931

[5] Mittler MA Walters BC Stopa EG. Observer reliability in histological grading of astrocytoma stereotactic biopsies. J Neurosurg. (1996) 85:1091–4. 10.3171/jns.1996.85.6.1091 8929500

[6] Prayson RA Agamanolis DP Cohen ML Estes ML Kleinschmidt-DeMasters BK Abdul-Karim F. Interobserver reproducibility among neuropathologists and surgical pathologists in fibrillary astrocytoma grading. J Neurol Sci. (2000) 175:33–9. 10.1016/S0022-510X(00)00274-4 10785254

[7] Castillo MS Davis FG Surawicz T Bruner JM Bigner S Coons S. Consistency of primary brain tumor diagnoses and codes in cancer surveillance systems. Neuroepidemiology. (2004) 23:85–93. 10.1159/000073980 14739573

[8] Cui Y Tha KK Terasaka S Yamaguchi S Wang J Kudo K. Prognostic imaging biomarkers in glioblastoma: development and independent validation on the basis of multiregion and quantitative analysis of MR images. Radiology. (2016) 278:546–53. 10.1148/radiol.2015150358 26348233 PMC4734164

[9] Sanghani P Ang BT King NKK Ren H. Overall survival prediction in glioblastoma multiforme patients from volumetric, shape and texture features using machine learning. Surg Oncol. (2018) 27:709–14. 10.1016/j.suronc.2018.09.002 30449497

[10] Henker C Kriesen T Glass Ä Schneider B Piek J. Volumetric quantification of glioblastoma: experiences with different measurement techniques and impact on survival. J Neurooncol. (2017) 135:391–402. 10.1007/s11060-017-2587-5 28755324

[11] Cuperlovic-Culf M Ferguson D Culf A Morin P Touaibia M. 1H NMR metabolomics analysis of glioblastoma subtypes: correlation between metabolomics and gene expression characteristics. J Biol Chem. (2012) 287:20164–75. 10.1074/jbc.M111.337196 22528487 PMC3370199

[12] Roda JM Pascual JM Carceller F González-Llanos F Pérez-Higueras A Solivera J. Nonhistological diagnosis of human cerebral tumors by 1H magnetic resonance spectroscopy and amino acid analysis. Clin Cancer Res. (2000) 6:3983–93. 11051247

[13] Opstad KS Wright AJ Bell BA Griffiths JR Howe FA. Correlations between *in vivo* (1)H MRS and *ex vivo* (1)H HRMAS metabolite measurements in adult human gliomas. J Magn Reson Imaging. (2010) 31:289–97. 10.1002/jmri.22039 20099340

[14] García-Martín ML Hérigault G Rémy C Farion R Ballesteros P Coles JA. Mapping extracellular pH in rat brain gliomas *in vivo* by 1H magnetic resonance spectroscopic imaging: comparison with maps of metabolites. Cancer Res. (2001) 61:6524–31. 11522650

[15] Vander Heiden MG Cantley LC Thompson CB. Understanding the Warburg effect: the metabolic requirements of cell proliferation. Science. (2009) 324:1029–33. 10.1126/science.1160809 19460998 PMC2849637

[16] Righi V Roda JM Paz J Mucci A Tugnoli V Rodriguez-Tarduchy G. 1H HR-MAS and genomic analysis of human tumor biopsies discriminate between high and low grade astrocytomas. NMR Biomed. (2009) 22:629–37. 10.1002/nbm.1377 19322812

[17] Glunde K Bhujwalla ZM Ronen SM. Choline metabolism in malignant transformation. Nat Rev Cancer. (2011) 11:835–48. 10.1038/nrc3162 22089420 PMC4337883

[18] Solivera J Cerdán S Pascual JM Barrios L Roda JM. Assessment of 31P-NMR analysis of phospholipid profiles for potential differential diagnosis of human cerebral tumors. NMR Biomed. (2009) 22:663–74. 10.1002/nbm.1387 19378301

[19] Hattingen E Raab P Franz K Lanfermann H Setzer M Gerlach R. Prognostic value of choline and creatine in WHO grade II gliomas. Neuroradiology. (2008) 50:759–67. 10.1007/s00234-008-0409-3 18523762

[20] Roldan-Valadez E Rios C Motola-Kuba D Matus-Santos J Villa AR Moreno-Jimenez S. Choline-to-N-acetyl aspartate and lipids-lactate-to-creatine ratios together with age assemble a significant Cox's proportional-hazards regression model for prediction of survival in high-grade gliomas. Br J Radiol. (2016) 89:20150502. 10.1259/bjr.20150502 27626830 PMC5124820

[21] Tolia M Verganelakis D Tsoukalas N Kyrgias G Papathanasiou M Mosa E. Prognostic value of MRS metabolites in postoperative irradiated high grade gliomas. Biomed Res Int. (2015) 2015:341042. 10.1155/2015/341042 26339606 PMC4538329

[22] Gao W Wang X Li F Shi W Li H Zeng Q. Cho/Cr ratio at MR spectroscopy as a biomarker for cellular proliferation activity and prognosis in glioma: correlation with the expression of minichromosome maintenance protein 2. Acta Radiol. (2019) 60:106–12. 10.1177/0284185118770899 29665708

[23] Shao W Gu J Huang C Liu D Huang H Huang Z. Malignancy-associated metabolic profiling of human glioma cell lines using 1H NMR spectroscopy. Mol Cancer. (2014) 13:197. 10.1186/1476-4598-13-197 25163530 PMC4158044

[24] Guidoni L Ricci-Vitiani L Rosi A Palma A Grande S Luciani AM. 1H NMR detects different metabolic profiles in glioblastoma stem-like cells. NMR Biomed. (2014) 27:129–45. 10.1002/nbm.3044 24142746

[25] Kleihues P Burger PC Scheithauer BW. The new WHO classification of brain tumours. Brain Pathol. (1993) 3:255–68. 10.1111/j.1750-3639.1993.tb00752.x 8293185

[26] Shinoda J Sakai N Murase S Yano H Matsuhisa T Funakoshi T. Selection of eligible patients with supratentorial glioblastoma multiforme for gross total resection. J Neurooncol. (2001) 52:161–71. 10.1023/A:1010624504311 11508816

[27] Matyja E Grajkowska W Stȩpień K Naganska E. Heterogeneity of histopathological presentation of pilocytic astrocytoma - diagnostic pitfalls. A review. Folia Neuropathol. (2016) 54:197–211. 10.5114/fn.2016.62530 27764513

[28] Collins VP Jones DT Giannini C. Pilocytic astrocytoma: pathology, molecular mechanisms and markers. Acta Neuropathol. (2015) 129:775–88. 10.1007/s00401-015-1410-7 25792358 PMC4436848

[29] Cerdàn S Parrilla R Santoro J Rico M. 1H NMR detection of cerebral myo-inositol. FEBS Lett. (1985) 187:167–72. 10.1016/0014-5793(85)81235-7 4018254

[30] Klunk WE Xu CJ Panchalingam K McClure RJ Pettegrew JW. Analysis of magnetic resonance spectra by mole percent: comparison to absolute units. Neurobiol Aging. (1994) 15:133–40. 10.1016/0197-4580(94)90153-8 8159259

[31] Govindaraju V Young K Maudsley AA. Proton NMR chemical shifts and coupling constants for brain metabolites. NMR Biomed. (2000) 13:129–53. 10.1002/1099-1492(200005)13:3<129::AID-NBM619>3.0.CO;2-V 10861994

[32] Breiman L Classification and Regression Trees. Belmont, CA: Wadsworth International Group (1984).

[33] Yamasaki F Kurisu K Kajiwara Y Watanabe Y Takayasu T Akiyama Y. Magnetic resonance spectroscopic detection of lactate is predictive of a poor prognosis in patients with diffuse intrinsic pontine glioma. Neuro Oncol. (2011) 13:791–801. 10.1093/neuonc/nor038 21653595 PMC3129271

[34] Steidl E Pilatus U Hattingen E Steinbach JP Zanella F Ronellenfitsch MW. Myoinositol as a biomarker in recurrent glioblastoma treated with bevacizumab: A 1H-magnetic resonance spectroscopy study. PLoS ONE. (2016) 11:e0168113. 10.1371/journal.pone.0168113 28033329 PMC5198997

[35] Hattingen E Bähr O Rieger J Blasel S Steinbach J Pilatus U. Phospholipid metabolites in recurrent glioblastoma: *in vivo* markers detect different tumor phenotypes before and under antiangiogenic therapy. PLoS ONE. (2013) 8:e56439. 10.1371/journal.pone.0056439 23520454 PMC3592858

[36] Quon H Brunet B Alexander A Murtha A Abdulkarim B Fulton D. Changes in serial magnetic resonance spectroscopy predict outcome in high-grade glioma during and after postoperative radiotherapy. Anticancer Res. (2011) 31:3559–65. 21965778

[37] Steffen-Smith EA Shih JH Hipp SJ Bent R Warren KE. Proton magnetic resonance spectroscopy predicts survival in children with diffuse intrinsic pontine glioma. J Neurooncol. (2011) 105:365–73. 10.1007/s11060-011-0601-x 21567301 PMC3199333

[38] Chang SM Nelson S Vandenberg S Cha S Prados M Butowski N. Integration of preoperative anatomic and metabolic physiologic imaging of newly diagnosed glioma. J Neurooncol. (2009) 92:401–15. 10.1007/s11060-009-9845-0 19357966 PMC2834319

[39] Li X Jin H Lu Y Oh J Chang S Nelson SJ. Identification of MRI and 1H MRSI parameters that may predict survival for patients with malignant gliomas. NMR Biomed. (2004) 17:10–20. 10.1002/nbm.858 15011246

[40] Reijneveld JC van der Grond J Ramos LM Bromberg JE Taphoorn MJ. Proton MRS imaging in the follow-up of patients with suspected low-grade gliomas. Neuroradiology. (2005) 47:887–91. 10.1007/s00234-005-1435-z 16133483

[41] Hattingen E Raab P Franz K Zanella FE Lanfermann H Pilatus U. Myo-inositol: a marker of reactive astrogliosis in glial tumors? NMR Biomed. (2008) 21:233–41. 10.1002/nbm.1186 17562554

[42] Jaskólski DJ Fortuniak J Majos A Gajewicz W Papierz W Liberski PP. Magnetic resonance spectroscopy in intracranial tumours of glial origin. Neurol Neurochir Pol. (2013) 47:438–49. 10.5114/ninp.2013.32999 24166565

[43] Leather T Jenkinson MD Das K Poptani H. Magnetic resonance spectroscopy for detection of 2-hydroxyglutarate as a biomarker for IDH mutation in gliomas. Metabolites. (2017) 7:E29. 10.3390/metabo7020029 28629182 PMC5488000

[44] Castillo M Smith JK Kwock L. Correlation of myo-inositol levels and grading of cerebral astrocytomas. AJNR Am J Neuroradiol. (2000) 21:1645–9. 11039343 PMC8174883

[45] Pascual JM Solivera J Prieto R Barrios L López-Larrubia P Cerdán S. Time course of early metabolic changes following diffuse traumatic brain injury in rats as detected by (1)H NMR spectroscopy. J Neurotrauma. (2007) 24:944–59. 10.1089/neu.2006.0190 17600512

[46] Croall I Smith FE Blamire AM. Magnetic resonance spectroscopy for traumatic brain injury. Top Magn Reson Imaging. (2015) 24:267–74. 10.1097/RMR.0000000000000063 26502308

[47] Kim JH Chang KH Na DG Song IC Kwon BJ Han MH. 3T 1H-MR spectroscopy in grading of cerebral gliomas: comparison of short and intermediate echo time sequences. AJNR Am J Neuroradiol. (2006) 27:1412–8. 16908549 PMC7977521

[48] Natsumeda M Igarashi H Nomura T Ogura R Tsukamoto Y Kobayashi T. Accumulation of 2-hydroxyglutarate in gliomas correlates with survival: a study by 3.0-tesla magnetic resonance spectroscopy. Acta Neuropathol Commun. (2014) 2:158. 10.1186/s40478-014-0158-y 25376594 PMC4236810

[49] Fan G Sun B Wu Z Guo Q Guo Y. *In vivo* single-voxel proton MR spectroscopy in the differentiation of high-grade gliomas and solitary metastases. Clin Radiol. (2004) 59:77–85. 10.1016/j.crad.2003.08.006 14697379

[50] Galanaud D Nicoli F Chinot O Confort-Gouny S Figarella-Branger D Roche P. Noninvasive diagnostic assessment of brain tumors using combined *in vivo* MR imaging and spectroscopy. Magn Reson Med. (2006) 55:1236–45. 10.1002/mrm.20886 16680716

[51] Opstad KS Ladroue C Bell BA Griffiths JR Howe FA. Linear discriminant analysis of brain tumour (1)H MR spectra: a comparison of classification using whole spectra versus metabolite quantification. NMR Biomed. (2007) 20:763–70. 10.1002/nbm.1147 17326043

[52] Sabatier J Gilard V Malet-Martino M Ranjeva JP Terral C Breil S. Characterization of choline compounds with *in vitro* 1H magnetic resonance spectroscopy for the discrimination of primary brain tumors. Invest Radiol. (1999) 34:230–5. 10.1097/00004424-199903000-00013 10084669

[53] Tsun ZY Possemato R. Amino acid management in cancer. Semin Cell Dev Biol. (2015) 43:22–32. 10.1016/j.semcdb.2015.08.002 26277542 PMC4800996

